# 560 A National Analysis of Discharge Disposition in Older Adults with Burns Estimating Independence at Discharge

**DOI:** 10.1093/jbcr/irac012.188

**Published:** 2022-03-23

**Authors:** Jason Cobert, Clifford C Sheckter, Tam N Pham

**Affiliations:** University of Rochester, Bellevue, Washington; Stanford University, Stanford, California; University of Washington, Seattle, Washington

## Abstract

**Introduction:**

Whereas older age strongly predicts higher burn mortality, the impact of age on discharge disposition is less well defined. Both providers and patients need a better understanding of the likelihood of discharge to non-independent living, as this factor may matter most to older patients. This investigation assesses the relationship between older age and discharge disposition after burns, in a nationally representative sample.

**Methods:**

We queried the 2007-2015 National Trauma Data Bank (NTDB) for burn hospitalizations in older adults. Pre-defined age categories were 55-64 years (working age group), 65-74 years (young-old), 75-84 years (middle-old), and 85+ years (old-old). Covariables included inhalation injury, comorbidities, burn total body surface area (TBSA), injury mechanism, and race. Discharge to non-independent living (nursing home, rehabilitation, and other facilities) was the primary outcome. Logistic regression was used to assess the association between older age and discharge to non-independent living, adjusting for covariables.

**Results:**

There were 25,840 burn hospitalizations in older adults with complete data during the study period. Working-age patients comprised 13,563 (53%) of admissions. Young-old accounted for 7,342 (28%), while middle-old comprised 3,876 (15%), and 1,059 (4%) were classified as old-old. Discharge to non-independent living steadily increased with burn TBSA and older age in survivors (Table). Beginning in the 65-74 age group (young-old), most patients with burns ≥20% TBSA were discharged to non-independent living. After adjusting for patient and injury factors, odd ratios for discharge to non-independent living were 2.1 for young-old, 3.5 for middle-old and 7.6 for old-old patients, when compared to working-age patients (all p< 0.001).

**Conclusions:**

Older age is a strong predictor of discharge to non-independent living after burns. These findings provide a realistic discharge framework for providers and older adults with acute burns.

